# Supplementary material for: Resistance to Gemcitabine in Pancreatic Cancer Is Connected to Methylglyoxal Stress and Heat Shock Response
Source: Cells. 2023 May 17;12(10):1414. doi: 10.3390/cells12101414 (PMC10217245; doi:10.3390/cells12101414)
Supplement: Supplementary file 1 [file cells-12-01414-s001.zip › cells-2384548-supplementary.pdf]

Supplementary

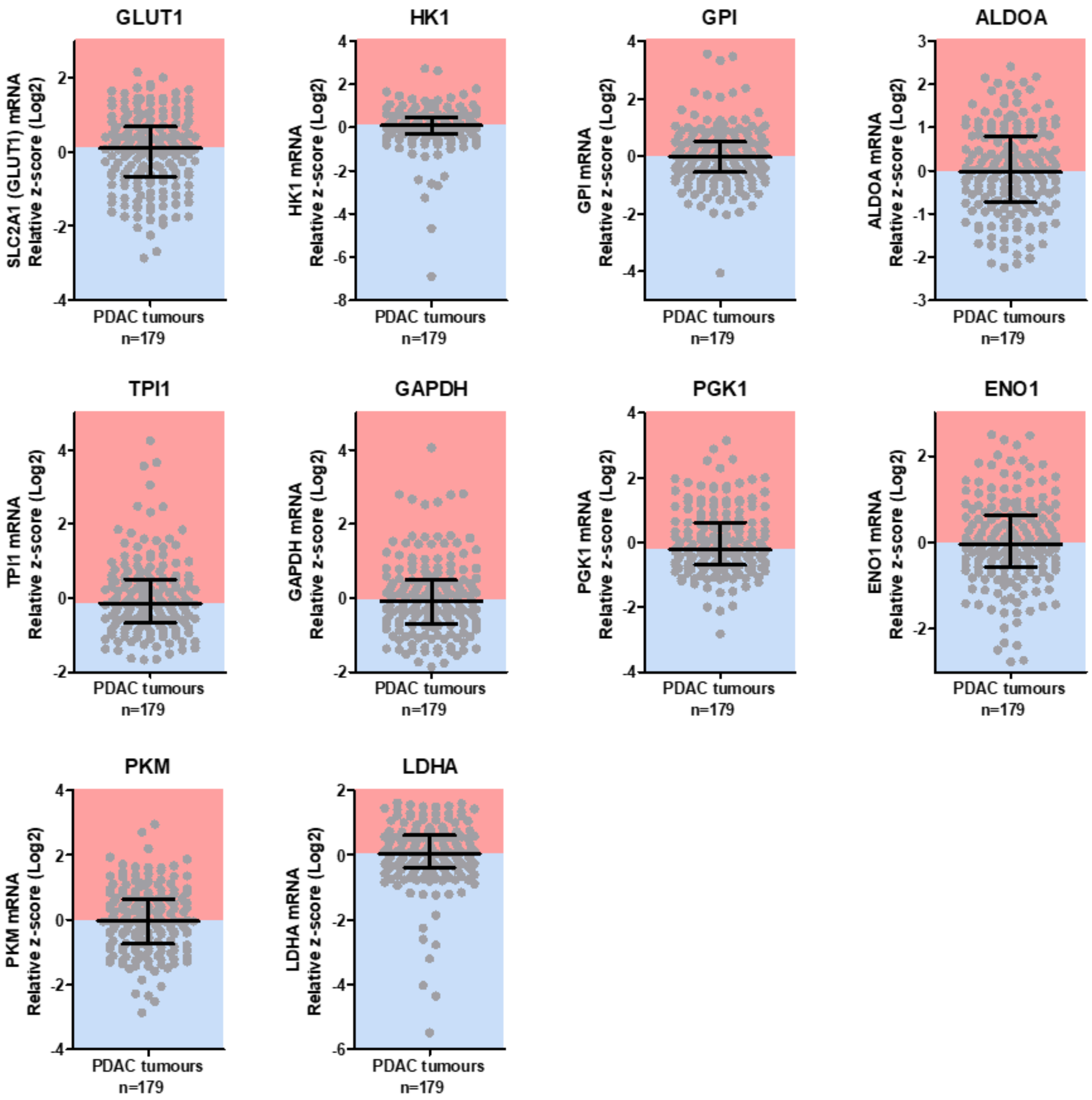

Figure S1. Gene expression of glycolysis enzymes (GLUT1, HK1, G6PD, ALDOA, TPI1, GAPDH, PGK1, ENO1, PKM and LDHA) split into high (red shading) and low (blue shading) based on median, for n=179 PDAC tumors (TCGA data).

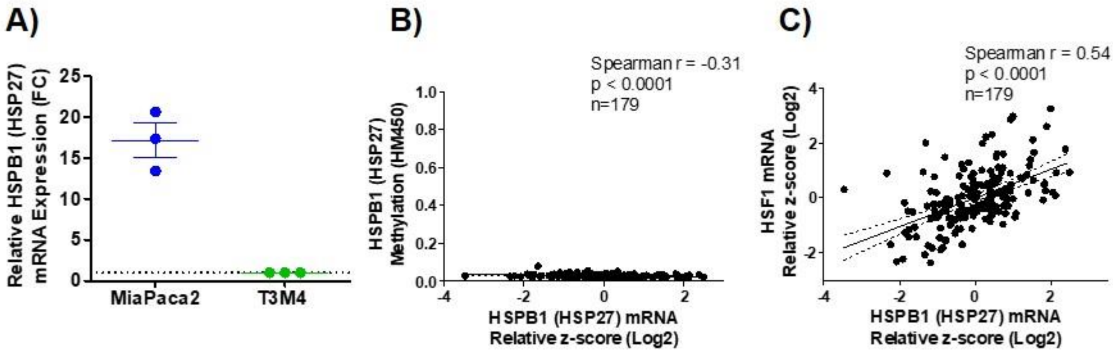

Figure S2. (A) mRNA levels of HSP27 (*HSPB1*) assessed by RT-qPCR in parental T3M4 and MiaPaca2 cells. mRNA levels are shown as relative to parental T3M4 cells and data were analyzed using paired t-test and shown as mean values ± SEM of three independent experiments; *$p < 0.05$. Correlation between HSP27 (*HSPB1*) mRNA levels and **(B)** HSP27 (*HSPB1*) gene methylation (HM450) and **(C)** *HSF1* mRNA levels across n=179 PDAC tumors (TCGA data). Data were analyzed by linear regression; statistical significance indicated.

Table S1. List of antibodies.

Antibody	Concentration	Company	Identifier
ArgP	1 in 10,000	Oya et al. 1999	Cat# mAb6B
β-actin	1 in 5,000	Sigma-Aldrich	Cat# A5441
GLO1	1 in 1,000	BioMAC	Cat# 02-14
GLUT1	1 in 1,000	Cell Signaling	Cat# 12939
HSF1	1 in 1,000	Cell Signaling	Cat# 12972
HSP27	1 in 3,000	Enzo Life Sciences	Cat# ADI-SPA-803-D
HSP90	1 in 1,000	Cell Signaling	Cat# 4877
LDHA	1 in 1,000	Cell Signaling	Cat# 3582
MGHs	1 in 1,000	Cell Biolabs	Cat# STA-011

Table S2. Oncogenic mutations investigated in parental and gemcitabine resistant cells.

Gene	Variant	Classification	Exon	Consequence	External IDs	Cell lines			
						T3M4		MiaPaca2	
						P	GR	P	GR
ALK	c.4587C>G (p.Asp1529Glu)	Benign	29 of 29	Missense Variant	rs1881421	+	+	+	+
ALK	c.4472A>G (p.Lys1491Arg)	Benign	29 of 29	Missense Variant	rs1881420	+	+	+	+
ALK	c.4381A>G (p.Ile1461Val)	Benign	29 of 29	Missense Variant	rs1670283	+	+	+	+
ALK	c.3375C>A	Benign	21 of 29	Synonymous Variant	rs3795850	+	+	+	+
PDGFRA	c.1701A>G	Benign	12 of 23	Synonymous Variant	rs1873778	+	+	+	+
PDGFRA	c.2472C>T	Benign	18 of 23	Synonymous Variant	rs2228230	+	+	-	-
MET	c.3912C>T	Benign	20 of 21	Synonymous Variant	rs41736	+	+	-	-
MET	c.534C>T	Benign	2 of 21	Synonymous Variant	rs35775721	-	-	+	+
HRAS	c.81T>C	Benign	2 of 6	Synonymous Variant	rs12628	+	+	+	+
DDR2	c.1260C>G	Probably Benign	12 of 19	Synonymous Variant	rs2298258	+	+	-	-
FGFR3	c.1959G>A	Probably Benign	14 of 18	Synonymous Variant	rs7688609	+	+	+	+
KIT	c.1621A>C (p.Met541Leu)	Probably Benign	10 of 21	Missense Variant	rs3822214	+	+	-	-
KIT	c.2586G>C	Probably Benign	18 of 21	Synonymous Variant	rs3733542	+	+	-	-
HIST1H3B	c.*10C>T	Probably Benign	1 of 1	3' UTR Variant	rs2213284	+	+	-	-
EGFR	c.2361G>A	Probably Benign	20 of 28	Synonymous Variant	rs1050171	-	-	+	+
KRAS	c.183A>C (p.Gln61His)	Pathogenic	3 of 6	Missense Variant	rs17851045	+	+	-	-
KRAS	c.34G>T (p.Gly12Cys)	Pathogenic	2 of 6	Missense Variant	rs121913530	-	-	+	+

+ Mutation is present; - Mutation is not present; P= parental; GR= gemcitabine resistant. Additional oncogenes investigated, no mutations evidenced: AKT1, BRAF, CDKN2A, CTNNB1, ERBB2, ERBB4, FGFR2, H3F3A, IDH1, IDH2, MAP2K1, NRAS, PIK3CA, PIK3R1, PTEN, STK11.
